# MR-guided focused ultrasound (MRgFUS) ablation for non-spinal osteoid osteoma treatment: a prospective multicenter evaluation

**DOI:** 10.1186/2050-5736-2-S1-A24

**Published:** 2014-12-10

**Authors:** D Geiger, A Napoli, A Conchiglia, A Bazzocchi, M Mastantuono, U Albisinni, C Masciocchi, C Catalano

**Affiliations:** 1 Sapienza University of Rome, Italy

## Background

Purpose of this study was to evaluate technical success, complications and one year follow-up clinical success for non-spinal painful osteoid osteomas treated with MR-guided Focused Ultrasound (MRgFUS).

## Materials and methods

This IRB approved prospective multicenter study was performed between May 2010 and April 2012. Thirty consecutive patients (M:21, F:9; Age range: 10-47; Mean age: 25±16) have been enrolled at three different university centers for non-spinal painful osteoid osteomas and treated (29 lesions) using MRgFUS (3.0-T/1.5-T GE Discovery MR 750/450, Milwaukee, USA equipped with InSightec ExAblate 2100, Tirat Carmel, ISR). Lesions were previously diagnosed on imaging basis, including ce-dynamic MR (Gd-BOPTA, Bracco, Milan, Italy). Mean number of sonications and energy deposition has been recorded. Technical success was evaluated immediately after treatment for complications (skin burn, neurovascular and tendon or ligament injuries). Twelve-month observation period followed to evaluate clinical success rate, recurrence and long-term complications. Clinical success was determined on the basis of pain reduction using visual analog scales (VAS); baseline and post-treatment scores statistical difference was calculated (paired T-test).

## Results

Twenty-nine non-spinal osteoid osteomas have been successfully treated using MR-gFUS. Mean sonication number was 7±3 and mean delivered acoustic energy 1180±736 J. At 12 month follow-up complete clinical success was observed in 26 patients (26/29, 90%; Mean VAS:0±0). Partial treatment was observed in 3 patients (3/29, 10%; Mean VAS: 5±0), whom were treated in the early phase of the learning curve and subsequently underwent CT-gRFA (2/3) or open surgery (1/3). At clinical evaluation, pain score values showed a significant reduction (p<0.001) between baseline (Mean VAS: 8±1) and post-treatment (Mean VAS: 1±2). No complications have been observed during, immediately after treatment and at one-year follow-up.

## Conclusion

Results of this prospective multicenter study, suggest that MR guided focused ultrasound may be considered an effective and safe alternative approach in non-spinal osteoid osteoma interventional management. Complete clinical success rate of 90% was demonstrated without adverse events.

